# Dietary flavonoid intakes and CVD incidence in the Framingham Offspring Cohort

**DOI:** 10.1017/S0007114515003141

**Published:** 2015-09-03

**Authors:** Paul F. Jacques, Aedin Cassidy, Gail Rogers, Julia J. Peterson, Johanna T. Dwyer

**Affiliations:** 1 Jean Mayer USDA Human Nutrition Research Center on Aging, Tufts University, Boston, MA 02111, USA; 2 The Friedman School of Nutrition Science and Policy, Tufts University, Boston, MA 02111, USA; 3 Department of Nutrition, Norwich Medical School, University of East Anglia, Norwich NR4 7UQ, UK; 4 Departments of Medicine and Community Health, Tufts University School of Medicine and Frances Stern Nutrition Center, Tufts Medical Center, Boston, MA 02111, USA

**Keywords:** CVD, Diet, Flavonoid intake, Observational study, Stroke

## Abstract

This study examines the relationship between long-term intake of six flavonoid classes and incidence of CVD and CHD, using a comprehensive flavonoid database and repeated measures of intake, while accounting for possible confounding by components of a healthy dietary pattern. Flavonoid intakes were assessed using a FFQ among the Framingham Offspring Cohort at baseline and three times during follow-up. Cox proportional hazards regression was used to characterise prospective associations between the natural logarithms of flavonoid intakes and CVD incidence using a time-dependent approach, in which intake data were updated at each examination to represent average intakes from previous examinations. Mean baseline age was 54 years, and 45 % of the population was male. Over an average 14·9 years of follow-up among 2880 participants, there were 518 CVD events and 261 CHD events. After multivariable adjustment, only flavonol intake was significantly associated with lower risk of CVD incidence (hazard ratios (HR) per 2·5-fold flavonol increase=0·86, *P*_trend_=0·05). Additional adjustment for total fruit and vegetable intake and overall diet quality attenuated this observation (HR=0·89, *P*_trend_=0·20 and HR=0·92, *P*_trend_=0·33, respectively). There were no significant associations between flavonoids and CHD incidence after multivariable adjustment. Our findings suggest that the observed association between flavonol intake and CVD risk may be a consequence of better overall diet. However, the strength of this non-significant association was also consistent with relative risks observed in previous meta-analyses, and therefore a modest benefit of flavonol intake on CVD risk cannot be ruled out.

In spite of many advances in the prevention and treatment of CVD and corresponding declines in deaths attributed to CHD and stroke, CVD remains the leading cause of death in the USA, accounting for 31·9 % of all deaths^(^ ^1^ ^)^. An estimated 620 000 experience incident CHD events and 610 000 incident strokes each year. Worldwide, it is estimated that there are more than seventeen million deaths each year due to CVD, accounting for 30 % of all deaths, and CVD incidence continues to increase in low- and middle-income countries where 80 % of CVD deaths occur^(^ ^2^ ^,^ ^3^ ^)^.

Diet has been shown to be a critical factor in CVD prevention^(^ ^4^ ^–^ ^6^ ^)^, and fruits and vegetables are an important part of diets associated with lower CVD risk^(^ ^7^ ^–^ ^10^ ^)^. However, the mechanism relating vegetable and fruit intake to CVD risk is still uncertain.

Dietary flavonoids – a class of polyphenols found in a large array of plant foods – have been examined as potential components associated with CVD risk. Previous studies have examined the relationship between flavonoid intakes and risk for CVD^(^ ^11^ ^)^, but many of these studies have one or more limitations – for example, the earlier studies examined only a few flavonoid classes. Length of follow-up was often limited, and in most cases with longer follow-up periods flavonoid intakes were based on a single baseline assessment, allowing the potential for regression dilution bias^(^ ^12^ ^,^ ^13^ ^)^. Finally, diets that are high in flavonoids are by their nature higher in fruits and vegetables and are generally of higher diet quality, but many studies did not account for possible confounding by overall diet quality, fruit and vegetable intake, fibre or other nutrients that might track with flavonoid intakes.

Therefore, we examined the relationship between incident CVD and CHD and long-term intake for individual flavonoid classes based on a more complete flavonoid database, and we also measured intakes repeatedly during follow-up while accounting for possible confounding by other components of a healthy diet pattern.

## Methods

### Population description

The Framingham Heart Study began in 1948 with the enrolment of 5209 adults aged 28–62 years, residing in Framingham, a town to the west of Boston, Massachusetts^(^ ^14^ ^)^, and has continued for over 60 years, with the survivors returning every 2 years for a physical examination and to complete a series of questionnaires as well as laboratory and cardiovascular tests. By 1971, the original cohort included 1644 husband–wife pairs and 378 individuals who had developed CVD. The offspring of these subjects and the spouses of the offspring were invited to participate, and 5135 of the 6838 eligible individuals participated in the first Framingham Offspring Study examination^(^ ^15^ ^)^. The Offspring Cohort undergoes repeat examination approximately every 3–4 years. For the present study, we used data derived from the fifth, sixth, seventh and eighth study examinations, which spanned 18 years (1991–2008), with follow-up for incident CVD through 2008.

To be eligible for the present study, participants had to attend the fifth study examination (baseline for the present study) and be free of CVD at baseline (*n* 3413). Of these eligible participants, we excluded 491 participants who were missing dietary data at baseline or at two consecutive follow-up examinations before the occurrence of a CVD event, censoring for other reasons (e.g. non-CVD death) or the end of follow-up (eighth examination). In addition, forty-two subjects were excluded because of missing data on critical baseline covariates. The final sample for this study (*N* 2880) included 1302 men and 1578 women.

This study was conducted according to the guidelines laid down in the Declaration of Helsinki, and all the procedures involving human participants were approved by the Boston University Medical Center Institutional Review Board. Written informed consent was obtained from all the participants. The present study was approved by the Tufts Medical Center Institutional Review Board.

### Dietary assessment

Dietary intakes were assessed using a validated semi-quantitative FFQ^(^ ^16^ ^)^ at the fifth, sixth, seventh and eighth examinations. The FFQ consisted of a list of foods with a standard serving size and a selection of nine frequency categories ranging from never or <1 serving/month to ≥6 servings/d. Participants were asked to report their frequency of consumption of each food item during the past year. Participants could also add up to three additional foods that are important components of their diets but were not listed in the questionnaire. Information on nutrient supplement use was also obtained by the FFQ. Dietary information was judged as unreliable and excluded from further analysis if reported total energy intakes were <2511 kJ/d (600 kcal/d) or >16 743 kJ/d (4000 kcal/d) for women and >17 580 kJ/d (4200 kcal/d) for men or if more than twelve food items were left blank.

The flavonoid database used for the FFQ was previously described^(^ ^17^ ^)^, and was primarily derived from the US Department of Agriculture flavonoid content of foods and the proanthocyanidin databases^(^ ^18^ ^,^ ^19^ ^)^. The same flavonoid database was used for all study examinations. We used the flavonoid classification of Cassidy *et al.* ^(^ ^17^ ^)^ (Table 1) to define the six flavonoid classes. Total flavonoids were defined as the sum of all six classes. We did not evaluate isoflavone intakes because habitual intakes are very low in the US diet^(^ ^20^ ^–^ ^23^ ^)^.

**Table 1:** Definition of flavonoid subclasses

Flavonoid subclass	Flavonoid compounds
Flavonols	Quercetin, kaempferol, myricetin, isorhamnetin
Flavones	Luteolin, apigenin
Flavanones	Eriodictyol, hesperetin, naringenin
Flavan-3-ols	Catechin, gallocatechin, epicatechin, epigallocatechin, epicatechin 3-gallate, epigallocatchin 3-gallate
Anthocyanidins	Cyanidin, delphinidin, malvidin, pelargonidin, petunidin, peonidin
Polymeric flavonoids	Proanthocyanidins (dimers, trimers, 4–6-mers, 7–10-mers, polymers, excluding monomers), theaflavins, thearubigins

The validity of flavonoid intake from the Harvard FFQ has not been directly assessed, but the validity for the food intake based on a comparison of the FFQ and two 7 d diet records collected during the year time interval covered by the FFQ has previously been published^(^ ^24^ ^)^. This comparison demonstrated relatively high correlation coefficients between intakes from the FFQ and diet records for the major dietary sources of flavonoids in the Framingham Offspring Cohort, including apples/pears (0·70), bananas (0·95), oranges (0·76), orange juice (0·78), strawberries (0·38), muffins (0·66), tea (0·77) and red wine (0·83).

### Ascertainment of CVD events and deaths

All the participants are under continuous surveillance for the occurrence of CVD events and death. Hospitalisation records and physician office visit records are obtained and reviewed by a committee of three experienced investigators. For each death, the same committee assigns an underlying cause of death based on Framingham Heart Study records, hospitalisation records and, when available, autopsy results^(^ ^25^ ^)^. Criteria for the diagnoses of cardiovascular events have been described elsewhere^(26)^. Incident CVD includes CHD (recognised or unrecognised myocardial infarction, angina pectoris, coronary insufficiency and CHD death), cerebrovascular disease (stroke and transient ischaemic attack), congestive heart failure (by Framingham criteria) and peripheral vascular disease (intermittent claudication). An unrecognised myocardial infarction was considered to have occurred if there was electrocardiographic evidence of significant loss of R waves or appearance of pathologic Q waves on serial tracings in the absence of a clinically recognised event. Sudden death, defined as death occurring within 1 h of onset of symptoms, was attributed to CHD, unless another cause was apparent^(^ ^25^ ^)^. Stroke was defined as an acute-onset focal neurological deficit of presumed vascular origin persisting for ≥24 h. Transient ischaemic attack was defined as an episode of rapid-onset focal neurological dysfunction attributed to focal cerebral ischaemia with resolution within 24 h. All cerebrovascular events were adjudicated by a panel of two neurologists^(27)^. For intermittent claudication, a physician-administered standardised questionnaire was used to elicit subjective symptoms of calf discomfort with exertion that occurred sooner with uphill or fast-paced walking and was alleviated with rest. All suspected claudication events were verified independently by a second physician examiner.

### Assessment of covariates

The covariates used in our analyses as potential confounders included sex, age, current smoking status, BMI, waist circumference, total energy intake, fruit and vegetable intake, overall diet quality, alcohol intake, hypertension, prevalent diabetes and cholesterol-lowering drug use. Medical history (e.g. medication use) and lifestyle activities (e.g. smoking history) were assessed during a standardised medical examination and interview. Waist circumference was measured at the level of the umbilicus at standing position. Height and weight were measured with the participant standing, with their shoes off and wearing only a hospital gown. BMI was calculated as body weight in kilograms divided by the square of height in metres. Alcohol intake was derived from the FFQ. Sitting blood pressure was measured twice for each participant after a 5 min rest using a random-zero sphygmomanometer, and the two readings were averaged for the analyses. Diabetes was defined as a plasma glucose level ≥7·0 mmol/l (fasting) or ≥11·1 mmol/l (non-fasting) or use of hypoglycaemic drug therapy. Overall dietary quality was assessed using the 2010 Dietary Guidelines for Americans Adherence Index (DGAI)^(^ ^28^ ^)^. The DGAI is a twenty-item score designed as a measure of adherence to the key recommendations from the 2005 Dietary Guidelines for Americans.

### Statistical methods

We used a time-dependent approach for the statistical analyses in which flavonoid intake data was updated at each examination as the cumulative mean of intakes from the current and all previous examinations – for example, events at the sixth examination were related to the mean of the intakes at the fifth and sixth examinations, events at the seventh examination were related to the mean of the intakes from the fifth, sixth and seventh examinations, etc. If participants were missing intake data at one of the follow-up examinations, the mean intake was based on the available intake data. Those who had data missing at baseline or two consecutive follow-up examinations before the development of an incident CVD event or the end of follow-up were excluded from the analyses. The natural logarithms of the different flavonoid class intakes were used as the exposure variables to account for non-linear relations with the outcomes total CVD and CHD incidence. We were substantially underpowered to consider stroke as a separate outcome (*n* 83). The time-dependent covariates (current smoking status, BMI, cholesterol-lowering medication use, diabetes and hypertension) were updated at each examination, and dietary covariates were updated as cumulative averages as we did for the flavonoid intakes. If covariates were missing at follow-up, they were carried forward from the previous examination.

Hazard ratios (HR) derived from Cox proportional hazards regression models (SAS PROC PHREG) were used to characterise the prospective associations between flavonoid intakes and incidence of CVD and CHD. As flavonoid intakes were transformed using a natural logarithm, the HR represents the relative risk for a proportional difference in flavonoid intake. We presented HR for a 2·5-fold difference in flavonoid intake, which approximated the smallest relative difference between the 75th and 25th percentile values for intake of the individual flavonoid classes across study examinations (Table 2).

**Table 2:** Flavonoid intake of members of the Framingham Heart Study Offspring Cohort at the fifth and eighth examinations (Medians and 25th, 75th percentiles; *n* 2880)

	Exam 5 (1991–1995)		Exam 8 (2005–2008)	
Flavonoid class	Median	25th, 75th percentiles	Median	25th, 75th percentiles
Flavonols (mg/d)	10·6	6·7, 16·5	11·7	7·5, 17·5
Flavones (mg/d)	1·6	0·8, 2·6	1·7	0·9, 2·7
Flavanones (mg/d)	35·1	11·0, 62·1	25·7	8·2, 57
Flavan-3-ols (mg/d)	21·7	11·8, 50·1	27·2	14·0, 59·5
Anthocyanins (mg/d)	9·1	3·4, 17·3	17·8	8·1, 29·2
Polymeric flavonoids (mg/d)	114	59, 226	156	86, 280
Total flavonoids* (mg/d)	212	124, 372	259	157, 436

* Total flavonoid intakes exclude isoflavones, which were not measured in this study.

For our analyses, we considered the following five different models: (1) an age- and sex-adjusted model (model 1); (2) a multivariable model including model 1 with additional adjustment for current smoking status, BMI and total energy intake (model 2); (3) model 2 with additional adjustment for fruit and vegetable intakes; (4) model 2 with additional adjustment for alcohol consumption; and (5) model 2 with additional adjustment for clinical covariates including waist circumference, hypertension, diabetes and cholesterol-lowering drug use. We also considered a model including variables in model 2 with additional adjustment for overall diet quality using the DGAI; however, given that the results were similar to the model with adjustment for fruit and vegetable intakes and the sample size available for the DGAI score was smaller due to missing data, we did not include our findings based on this model in our tables, but reported relevant findings in the text.

To examine the relationship between the top food sources of the specific flavonoid classes inversely associated with CVD, we identified all foods that contributed at least 10 % of the intake of each flavonoid class and related the intake of these foods to CVD incidence. We classified food intake into three categories, which were equivalent (<1 serving/week, 1–4 servings/week and ≥5 servings/week) and applied the same proportional hazards regression analysis approach that was described above for the flavonoids. A test for trend across food serving categories was based on assigning the median servings in each category to individuals in that category and treating that resulting variable as a continuous variable in the regression models.

To assess the validity of the proportional hazards assumption, we introduced interaction terms between follow-up time and our exposures, and we used likelihood ratio tests to assess evidence for departures from this assumption. There was no evidence that this assumption was violated for any of our models. We also performed a sensitivity analysis excluding CVD and CHD cases that occurred within the first 2 years of follow-up.

All the analyses were performed using SAS version 9.2 (SAS Institute). A *P* value <0·05 was considered statistically significant.

## Results

Median total flavonoid intakes (excluding isoflavones) increased modestly across follow-up from 212 mg/d at the fifth Framingham Offspring Study examination (1991–1995) to 259 mg/d at the eighth study examination (2005–2008) (Table 2). This increase was in large part due to higher intakes of polymeric flavonoids, which comprised more than half of the total flavonoids. Median anthocyanin intake also nearly doubled over this time period from 9·1 to 17·8 mg/d. There was substantial variation in intakes among participants as demonstrated by the interquartile ranges (75th–25th percentile values) of the total flavonoids and the flavonoid classes. In all cases, except for flavonol intake, the 75th percentile values were more than three times greater than the 25th percentile values, ranging to more than five to seven times higher for flavanones. For flavonols, the relative difference between the 75th and 25th percentiles was 2·5 at the fifth examination and 2·3 at the eighth examination.

Over an average of 14·9 years of follow-up (range 1 month–19·9 years), there were 518 incident CVD events, including 261 CHD events (112 myocardial infarctions, 102 angina pectoris, twenty CHD deaths, thirteen coronary insufficiency and fourteen unspecified), eighty-three strokes, forty-seven transient ischaemic attacks, seventy-nine congestive heart failure events, forty-four peripheral vascular disease events and four other/unspecified CVD events. Individuals with the highest total flavonoid intakes were more likely to be women and less likely to be smokers and to have lower BMI, higher energy, fruit and vegetable intakes and a better overall diet quality (Table 3). Prevalence of diabetes and hypertension and use of medications for hypercholesterolaemia, hypertension and diabetes were unrelated to flavonoid intake.

**Table 3:** Participant characteristics* by quartile categories of total flavonoid intake (Mean values and 95 % confidence limits (CL); or percentages)

	Overall (*n* 2880)		Quartile 1 (*n* 720)		Quartile 2 (*n* 720)		Quartile 3 (*n* 720)		Quartile 4 (*n* 720)		
Participant characteristics	Mean	95 % CL	Mean	95 % CL	Mean	95 % CL	Mean	95 % CL	Mean	95 % CL	*P*
Male (%)	45·2	43·4, 47	49·7	46·1, 53·4	49·4	45·8, 53·1	47·2	43·6, 50·8	34·4	30·8, 38	<0·001
Age (years)	54·2	53·8, 54·5	53·4	52·7, 54·1	54·3	53·6, 55	54·4	53·7, 55·1	54·7	54, 55·4	0·04
BMI (kg/m^2^)†	26·9	26·7, 27·0	27·4	27·1, 27·8	27·1	26·8, 27·5	26·7	26·3, 27·0	26·3	26·0, 26·6	<0·001
Current smoker (%)	18·3	16·9, 19·7	29·4	26·6, 32·2	17·3	14·5, 20·1	12·9	10·1, 15·7	13·8	11, 16·5	<0·001
Diabetes (%)	6·0	5·1, 6·9	6·7	5, 8·5	5	3·2, 6·7	7	5·2, 8·7	5·4	3·7, 7·1	0·55
Hypertension (%)	19·5	18·2, 20·9	19·7	16·9, 22·5	19·9	17·1, 22·7	20·3	17·5, 23·1	18·3	15·5, 21·1	0·42
Medication use											
Cholesterol-lowering (%)	5·3	4·5, 6·1	5·2	3·6, 6·8	4·8	3·2, 6·4	6·3	4·7, 7·9	4·9	3·3, 6·6	0·96
Anti-diabetic (%)	2·5	2, 3·1	2·8	1·7, 4	2·1	1, 3·3	2·4	1·3, 3·6	2·7	1·6, 3·9	0·87
Hypertension (%)	16·7	15·4, 18	17	14·4, 19·6	17·2	14·6, 19·8	18	15·4, 20·6	14·6	12, 17·3	0·16
Any at baseline (%)‡	19·7	18·3, 21·1	19·9	17·1, 22·7	19·5	16·8, 22·3	21·7	18·9, 24·5	17·6	14·8, 20·4	0·26
Any at follow-up (%)‡	58·1	56·4, 59·8	58·6	55·2, 62·1	58·9	55·5, 62·3	58·9	55·5, 62·3	55·9	52·4, 59·4	0·21
Energy (kJ/d)†	7476·8	7388·9, 7564·7	6146·3	6008·2, 6284·4	7355·5	7192·3, 7518·6	8008·2	7832·4, 8188·1	8631·6	8439·1, 8824·1	<0·001
Energy (kcal/d)†	1787	1766, 1808	1469	1436, 1502	1758	1719, 1797	1914	1872, 1957	2063	2017, 2109	<0·001
Fruit intake (g)§	256	249, 263	128	120, 137	244	233, 256	336	323, 350	350	336, 365	<0·001
Vegetable intake (g)§	255	251, 260	224	216, 233	249	240, 257	273	264, 282	278	269, 287	<0·001
Diet quality (DGAI)	58·4	58, 58·8	52	51·3, 52·7	58	57·3, 58·7	61·8	61·1, 62·5	61·8	61·1, 62·5	<0·001

DGAI, Dietary Guidelines for Americans Adherence Index.

* Characteristics as measured at baseline, except as noted.

† Geometric means.

‡ Lipid-lowering, anti-diabetic or blood pressure-lowering medication use at baseline and at the last follow-up visit for each participant.

§ Square root transformation, back transformed to original units.

We observed that intakes of total flavonoids and all flavonoid classes, except for flavones and flavanones, were inversely associated with CVD incidence after adjusting for age and sex (Table 4). After additional adjustment for smoking, BMI and total energy intake, only flavonol intake remained significantly inversely associated with CVD incidence. Addition of waist circumference, hypertension, diabetes and cholesterol-lowering drug use to the previous model did not materially affect the association between flavonol and CVD incidence (HR=0·86, 95 % confidence limits (CL) 0·74, 1·01, *P*_trend_=0·06) and additional adjustment for alcohol intake modestly strengthened the association (HR=0·81, 95 % CL 0·68, 0·95, *P*_trend_=0·01). However, additional adjusting for fruit and vegetable intakes attenuated this relationship (Table 4). Additional adjustment for overall diet quality also attenuated the relationship (HR=0·92, 95 % CL 0·78, 1·09, *P*_trend_=0·33).

**Table 4:** Relative risk (RR)of total CVD events over 20 years of follow-up* for each 2·5-fold increase in daily flavonoid intake (Relative risks and 95 % confidence limits (CL))

	Age and sex adjusted			Multivariable adjusted†			Multivariable+fruit/vegetable		
Flavonoid class	RR	95 % CL	*P*	RR	95 % CL	*P*	RR	95 % CL	*P*
Flavonols	0·79	0·69, 0·91	0·001	0·86	0·74, 1·00	0·05	0·89	0·75, 1·06	0·20
Flavones	0·87	0·76, 1·00	0·06	0·98	0·84, 1·13	0·74	1·00	0·82, 1·21	0·99
Flavanones	0·95	0·89, 1·02	0·13	1·00	0·93, 1·07	0·98	1·00	0·92, 1·09	0·97
Flavan-3-ols	0·91	0·83, 0·98	0·02	0·95	0·88, 1·04	0·29	0·97	0·88, 1·05	0·43
Anthocyanins	0·89	0·82, 0·97	0·006	0·94	0·86, 1·02	0·14	0·95	0·86, 1·05	0·30
Polymeric flavonoids	0·87	0·80, 0·95	0·002	0·92	0·84, 1·01	0·08	0·93	0·84, 1·02	0·13
Total flavonoids	0·86	0·77, 0·95	0·004	0·93	0·83, 1·04	0·20	0·93	0·82, 1·06	0·26

* Mean follow-up time of 14·9 years; *n* 518 CVD events.

† Multivariable model adjusted for age (years), sex, current smoking status (yes/no), BMI (kg/m^2^) and total energy intake (kJ/d (kcal/d)).

Although in many cases the HR for the associations between flavonoid intakes and CHD incidence were similar to those for CVD incidence, only the inverse association between flavone intake and CHD incidence was statistically significant in the age- and sex-adjusted models, but was not statistically significant after adjusting for current smoking, BMI and total energy intake (Table 5). Addition of waist circumference, hypertension, diabetes and cholesterol-lowering drug use, alcohol intake or fruit and vegetable intakes to the previous model did not affect any of the associations between the flavonoids and CHD risk (data not shown).

**Table 5:** Relative risk (RR) of CHD events over 20 years of follow-up* for each 2·5-fold increase in daily flavonoid intake (Relative risks and 95 % confidence limits (CL))

	Age and sex adjusted			Multivariable adjusted†			Multivariable+fruit/vegetable		
Flavonoid class	RR	95 % CL	*P*	RR	95 % CL	*P*	RR	95 % CL	*P*
Flavonols	0·84	0·69, 1·02	0·08	0·90	0·73, 1·12	0·36	0·96	0·76, 1·22	0·76
Flavones	0·80	0·66, 0·97	0·03	0·87	0·71, 1·07	0·19	0·83	0·64, 1·09	0·18
Flavanones	0·92	0·84, 1·01	0·09	0·96	0·87, 1·06	0·40	0·94	0·85, 1·06	0·32
Flavan-3-ols	0·95	0·84, 1·07	0·38	1·00	0·88, 1·13	0·97	1·01	0·89, 1·14	0·86
Anthocyanins	0·92	0·82, 1·03	0·14	0·96	0·85, 1·08	0·49	0·98	0·86, 1·12	0·76
Polymeric flavonoids	0·91	0·8, 1·03	0·13	0·96	0·84, 1·10	0·53	0·97	0·84, 1·12	0·68
Total flavonoids	0·88	0·76, 1·02	0·10	0·95	0·81, 1·11	0·51	0·96	0·80, 1·14	0·63

* Mean follow-up time of 14·9 years; *n* 261 CHD events.

† Multivariable model adjusted for age (years), sex, current smoking status (yes/no), BMI (kg/m^2^) and total energy intake (kJ/d (kcal/d)).

We performed sensitivity analyses excluding thirty-three incident cases of CVD and twenty-two incident cases of CHD that occurred within the first 2 years of follow-up. Findings from these analyses were similar to the complete analyses, and therefore the results are not presented.

As flavonol intake remained significantly associated with CVD incidence after multivariable adjustment, we examined CVD incidence by intake categories of the top food sources of flavonols (Table 6). Tea and apples/pears were the only individual food items that contributed at least 10 % of total flavonol intake and together comprised approximately 31 % of flavonol intake at the fifth Offspring examination. Although both tea and apple/pear consumption were inversely associated with CVD incidence after adjusting for age and sex, both associations were attenuated after multivariable adjustment. The association with tea remained marginally significant (*P*_trend_=0·07).

**Table 6:** Relation of cumulative food intake and total CVD events over 20 years of follow-up* for the Framingham Heart Study Offspring Cohort (Hazard ratios (HR) and 95 % confidence limits (CL))

	Age and sex adjusted			Multivariable adjusted†		
Food (amount/week)‡	HR	95 % CL	*P*	HR	95 % CL	*P*
Tea (ml)						
<237	1			1		
237–948	0·91	0·75, 1·10	0·34	1·00	0·82, 1·21	0·98
1185+	0·71	0·55, 0·92	0·01	0·78	0·60, 1·01	0·07
*P*_trend_			0·01			0·07
Apples/pears (g)						
<138	1			1		
138–118	0·75	0·58, 0·97	0·03	0·82	0·63, 1·06	0·14
148+	0·62	0·42, 0·91	0·02	0·74	0·50, 1·10	0·14
*P*_trend_			0·03			0·16

* Mean follow-up time of 14·9 years; *n* 518 CVD events.

† Multivariable model adjusted for age (years), sex, current smoking status (yes/no), BMI (kg/m^2^) and total energy intake (kJ/d (kcal/d)).

‡ The categories of food intake amounts are equivalent to <1 serving/week, 1–4 servings/week and ≥5 servings/week, respectively, with one serving defined as 8 fl. oz. of tea and one medium apple or pear.

## Discussion

Our findings, based on long-term follow-up of middle-aged and older Americans, suggest a possible benefit of a higher flavonol intake on risk of CVD, but this may in part be due to differences in dietary patterns among those consuming the highest amounts of flavonols, such as higher fruit and vegetable intake. Existing evidence regarding flavonoid intake and CVD risk remains largely equivocal^(^ ^11^ ^)^. Two recent meta-analyses from the same research group including fifteen prospective studies (representing eleven unique cohorts, fourteen in a meta-analysis of CVD events^(^ ^29^ ^)^ and eight in a meta-analysis of stroke events^(^ ^30^ ^)^) concluded that dietary intakes of all six flavonoid classes were associated with lower risks of CVD and flavonol intake was associated with a lower stroke risk in men.

However, many of the existing concerns with the previous epidemiological studies, such as inclusion of all classes in the flavonoid databases, the possible confounding by diet quality and the use of a single baseline estimate of intake, are not addressed in these meta-analyses^(^ ^29^ ^,^ ^30^ ^)^. For example, eleven of these fifteen studies used older flavonoid databases that included only a few flavonoid classes. Four of these studies considered only one class of flavonoids^(^ ^31^ ^–^ ^34^ ^)^ and seven considered only two to three classes^(^ ^35^ ^–^ ^41^ ^)^.

None of these fifteen studies considered confounding by overall diet quality, three considered possible confounding by a single dietary factor that may be higher in flavonoid-containing foods such as dietary fibre or vitamin C^(^ ^31^ ^–^ ^33^ ^)^, and only one study considered potential confounding by fruit and vegetable intake^(^ ^40^ ^)^. Mink *et al.* ^(^ ^42^ ^)^ included the most complete adjustment of dietary factors (indirectly addressing overall diet quality), including intakes of whole grains, fish and seafood, folate, vitamin C, *β*–carotene and fatty acids in their analyses.

The longer length of follow-up and assessment of diet only at baseline combine to increase the probability of regression dilution bias, which results from misclassification of exposure using only baseline values^(^ ^12^ ^,^ ^13^ ^)^. Regression dilution bias results in the attenuation of the true association. To prevent such bias, replicate measures are needed during follow-up. Only two of these studies collected replicate measures of flavonoid intakes during follow-up^(^ ^41^ ^,^ ^43^ ^)^. Of the thirteen that relied only on baseline assessment to characterise flavonoid intakes^(^ ^31^ ^–^ ^40^ ^,^ ^42^ ^,^ ^44^ ^,^ ^45^ ^)^, the average follow-up time was 10·8 years, allowing ample time for variation in flavonoid intakes.

Potential limitations for the existing studies described above will not similarly explain a lack of association resulting from bias or the existence of an apparent association due to bias. For example, given that flavonoids are found in plant-based foods, the overall diet quality of higher flavonoid consumers is likely to be better than those consuming lower amounts of flavonoids, as we have shown in the present study. Foods containing greater amounts of flavonoids tend to be good sources of nutrients (such as K, Mg, fibre and lignans) that might reduce CVD risk and lower in other nutrients (such as SFA and Na) that may increase CVD risk. Consequently, failure to account for potential confounding by overall dietary quality or other specific dietary factors that may be associated with flavonoid intakes would be a likely cause of false-positive associations. The use of incomplete flavonoid databases and the combination of long follow-up periods without accounting for possible changes in exposure, on the other hand, would be more likely to diminish the strength of any true associations resulting in false-negative associations. Consequently, these sources of potential bias would not likely be responsible for the observed associations between flavonoids and CVD risk summarised in the aforementioned meta-analyses^(^ ^29^ ^,^ ^30^ ^)^.

When we attempted to address these limitations in the present study, the only observation that remained statistically significant after adjusting for age, sex, BMI, current smoking and total energy intake was the inverse relationship between flavonol intakes and CVD. This association was attenuated and no longer statistically significant after further adjustment for fruit and vegetable consumption or overall diet quality, suggesting that this relation was due, at least in part, to better diets consumed by individuals with higher flavonol intakes. However, even after adjusting for fruits and vegetables, the observed relative risk was 0·89, which was identical to the overall relative risk (0·89, 95 % CL 0·84, 0·94) reported by Wang *et al.* ^(^ ^29^ ^)^ in their meta-analysis of flavonols and CVD risk.

Several mechanisms have been proposed by which flavonols might influence CVD risk, including effects on endothelium and vascular smooth muscle, platelet function, blood pressure, inflammation, body weight and composition, and insulin resistance^(^ ^46^ ^,^ ^47^ ^)^. Flavonols are widely distributed in plant products. In the Framingham Offspring Cohort, we have previously show that the major sources of dietary flavonols were tea, apples and pears, together constituting about 31 % of flavonol intake^(^ ^48^ ^)^. We observed that CVD incidence was inversely associated with consumption of both tea and apples and pears, but these associations were attenuated and no longer statistically significant after additional adjustments for smoking, BMI and total energy intake. However, even the attenuated HR indicated a >20 % lower CVD risk for the highest consumers of both of these food items.

We had previously observed associations between flavonol and flavan-3-ol intakes and type 2 diabetes, an important CVD risk factor^(^ ^48^ ^)^. Consequently, the weak association between flavonol and CVD and the absence of an association between flavan-3-ols, with or without accounting for type 2 diabetes in this population, is puzzling. As a part of the longest longitudinal study of CVD development in the USA, the Framingham Offspring Cohort and their healthcare providers may be uniquely attuned to CVD risk. It is possible that the large proportion of the population on medications for CVD risk factors (including antihypertensive, cholesterol lowering and diabetes medications) may have limited our ability to detect any association with flavonoids. Although the use of these medications was relatively low at baseline, 58 % of the participants were taking one or more of these medications by the end of their follow-up. The use of medications may have effects on disease risk that are stronger than dietary influences and may have diminished the ability to detect associations between dietary factors and CVD risk.

The usual doses of flavonoids used in intervention studies examining their effects on CVD risk factors tend to be many times greater than the intakes of flavonoids in most populations. For flavonols, the intervention doses are usually at least ten times greater^(^ ^49^ ^)^ than those seen in the Framingham Offspring Cohort. The non-linear nature of the relationship we observed between flavonol intake and CVD risk suggest that benefits may be achieved with lower doses over a longer time period. Our models suggest that the benefit of increasing flavonol dose on CVD risk diminishes in an exponential manner, with most of the benefit obtained by modest increases among individuals with the lowest intakes. For example, a 2·5-fold difference in intake from about 4 to 10 mg/d (or change of 6 mg/d) is associated with a 14 % reduction in CVD risk in our multivariable model, whereas a similar reduction in risk would be seen for a 30 mg/d difference for those with intakes of 20 mg/d.

Our study accounted for many limitations of earlier studies – for example, we used more complete flavonoid databases, our analyses accounted for overall dietary quality and intake of important sources of flavonoids (fruits and vegetables) and we used multiple assessments of flavonoid intake collected during follow-up. However, this work still has several limitations. We used the Harvard FFQ to assess flavonoid intake, which may not have captured intakes of some of the richer, but less frequently consumed, sources of flavonoids, such as cranberries, spices (dill, thyme, mint), nuts (pecans, walnuts), beans (black, kidney, fava). However, the Harvard FFQ does well for many of the most frequently consumed flavonoid sources^(^ ^24^ ^)^ including apples, bananas, oranges and orange juice, tea and red wine. Another limitation is that the most recent version of the USDA Database for the Flavonoid Content of Selected Foods^(^ ^50^ ^)^ had not been incorporated into the Harvard FFQ database at the time we performed our analyses. In addition, we recognise that the chemical analyses currently available for assessing thearubigin intakes represent only a rather crude estimate; however, for completeness, we wanted to include all available flavonoid intake data^(^ ^51^ ^)^. Although CHD accounted for more than one-half of all incident CVD events, we did not see any statistically significant associations after multivariable adjustment. This may be in part due to the lower power of the CHD analyses, as the relative risks, although NS, were consistent in magnitude with many of those reported in the earlier meta-analysis for overall CVD^(^ ^29^ ^)^. We also had insufficient power to examine the associations with stroke incidence in this population. The members of the Framingham Offspring Cohort are largely Caucasians of Northern European descent, and they and their healthcare providers are likely to be highly conscious of cardiovascular risk factors, and thus generalisations to other populations may be limited. Finally, we did not adjust for multiple testing because of hypothesised relations based on existing evidence.

In summary, the role that flavonoids may play in prevention of CVD remains uncertain. Our findings provide limited support for a beneficial relationship between a higher intake of flavonols and CVD incidence, but for none of the other flavonoid classes commonly consumed in the US diet. In spite of continuing improvements in the flavonoid databases and our dietary assessment tools, there is still a large amount of imprecision in our estimates of flavonoid intakes that limit our ability to determine their role in CVD and other health outcomes. Many methodological issues need to be addressed to allow us to more adequately examine these associations^(^ ^52^ ^)^. In the meantime, animal experiments and clinical intervention trials with validated surrogate outcomes of CVD will be essential to complement the limitations of the evidence from observational studies.
